# Experimental Germ Tube Induction in* Candida albicans*: An Evaluation of the Effect of Sodium Bicarbonate on Morphogenesis and Comparison with Pooled Human Serum

**DOI:** 10.1155/2017/1976273

**Published:** 2017-06-05

**Authors:** Tapiwa Matare, Pasipanodya Nziramasanga, Lovemore Gwanzura, Valerie Robertson

**Affiliations:** ^1^Department of Medical Laboratory Sciences, University of Zimbabwe, College of Health Sciences, Box A178, Harare, Zimbabwe; ^2^Department of Medical Microbiology, University of Zimbabwe, College of Health Sciences, Box A178, Avondale, Harare, Zimbabwe

## Abstract

**Objective:**

The potential of NaHCO_3_ versus human serum to induce germ tube formation in* Candida albicans* was investigated.

**Specimens:**

A total of 100 isolates were obtained from oral swabs of patients presenting with thrush. Approval for the study was granted by the Joint Research Ethics Committee (JREC/23/08).

**Method:**

Confirmed* C. albicans* isolates by routine methods were tested for germ tube induction using 5 different concentrations of Tris-maleate buffered NaHCO_3_ and Tris-maleate buffer control. Standard control strains included were* C. albicans* (ATCC 10231) and* C. krusei* (ATCC 6258). Microculture was done in 20 *μ*L inoculums on microscope slides for 3 hours at 37°C. The rate of germ tube formation at 10-minute intervals was determined on 100 isolates using the optimum 20 mM Tris-maleate buffered NaHCO_3_ concentration. Parallel germ tube formation using human serum was done in test tubes.

**Results:**

The optimum concentration of NaHCO_3_ in Tris-maleate buffer for germ tube induction was 20 mM for 67% of isolates. Only 21% of isolates formed germ tubes in Tris-maleate buffer control. There was no significant difference in induction between human serum and Tris-maleate buffered NaHCO_3_.

**Conclusion:**

Tris-maleate buffered NaHCO_3_ induced germ tube formation in* C. albicans* isolates at rates similar to human serum.

## 1. Introduction 

*Candida albicans* is an important pathogen among yeasts associated with other chronic infections and noninfectious disease conditions [1, 2].* C. albicans* can grow as budding yeast cells or as filamentous hyphal forms (mycelial state) depending on the growth conditions [3, 4]. As a commensal organism,* C. albicans* can be recovered from the hospital environment and as normal biota of the vagina, mouth, bowel, and skin of humans [1, 4]. It assumes a pathogenic role whenever the normal host defense mechanisms against disease are immunocompromised [5]. Mucocutaneous candidiasis occurs commonly where there is continuous dampness and poor hygiene of the skin or affected membranes. Oral thrush, vaginal thrush, and esophagitis are associated with HIV/AIDS, pregnancy, diabetes mellitus, and prolonged broad-spectrum antibiotic use [2, 3].

It is understood that the immunocompromised host's physiological conditions can induce dimorphism in* C. albicans* to a hyphal state of growth. These growth conditions are simulated in the laboratory whereby* C. albicans* grows as yeast only or short term germ tubes (hyphal state) [6–8]. This has been achieved by manipulating type and concentrations of carbohydrates and amino acids in culture media [4, 6, 8, 9].* C. albicans* grows as creamy-white pasty colonies with distinct budding yeasts on Sabouraud Dextrose Agar at 37°C. The hyphae are the predominant form with characteristic chlamydospores upon 24–48 hours' incubation at 30°C on cornmeal agar or rice agar. Short germ tubes are induced in rabbit serum or human serum at 37°C in the laboratory diagnostic tests to identify* C. albicans* from other species [6–8].

Endotrophic germ tube formation is the endogenous germination of* C. albicans* yeast cells. The germ tube has parallel walls and no constriction at the point of origin at the blastospore mother cell. It has been suggested to be a contributory virulence factor in the pathogenesis of* C. albicans* [3, 4, 9].

The use of human serum routinely for culture and microscopic examination of* C. albicans* in the germ tube test is cheap but presents a hazard for transmission of disease [3, 6]. Rabbit or sheep serum is safer but expensive to purchase commercially for countries with limited resources. Germ tube formation is linked to an increase in endogenous CO_2_ concentrations within the yeast cells. This study investigated the possibility of using sodium bicarbonate as an alternative to human serum for germ tube testing [6–8, 10, 11].

## 2. Material and Methods

### 2.1. Study Design

This was an experimental study whereby oral swabs would be collected from patients visiting an Opportunistic Infections Clinic (OI Clinic). Oral swabs would be cultured for* C. albicans* with subsequent species identification using routine laboratory methods. Experimental germ tube induction was also a laboratory designed adaptation of the routine microscope slide culture of yeasts for chlamydospore production.

### 2.2. Isolates

Ethics approval was sought and obtained from the Joint Parirenyatwa/College of Health Sciences Ethics Committee (JREC Approval number JREC/23/08). A total of one hundred and fifty (150) oral swabs were collected from patients at Wilkins Infectious Diseases Hospital's Opportunistic Infections Unit (OI Unit) during a 4-month period. The swabs were placed in transport media and taken to the Department of Medical Laboratory Sciences at the University of Zimbabwe, College of Health Sciences.

### 2.3. Culture

Swabs were cultured on Sabouraud Dextrose Agar (SDA) containing 1% chloramphenicol at 37°C for 48 hours. Colonies appearing visually as yeast were confirmed by Gram staining and microscopic examination. Isolates suspected of being* Candida albicans* were confirmed by the germ tube formation method using foetal bovine serum. Isolates confirmed to be* C. albicans* were suspended in sterile distilled water for storage. At the end of the sampling period, the stored isolates were subcultured on SDA medium and maintained at 20°C. Colonies were resuspended in sterile distilled water and washed five times by centrifugation at 3000 RPM for 15 minutes. After washing, the yeast cells were resuspended in 5 mL sterile deionized water. Microscopic examination for nonspecific pretest budding of yeast before use was done.

### 2.4. Bicarbonate Slide Technique

Tris-buffered sodium bicarbonate at 5 different concentrations (10 mM, 20 mM, 30 mM, 40 mM, and 50 mM) was tested for ability to induce germ tube production in 10 different isolates. The slide cultures were prepared in triplicate for each bicarbonate concentration.* C. albicans* strain ATCC 10231 and* C. krusei* ATCC 6258 were used as positive and negative controls, respectively. Another set of tubes containing 5 mL of Tris-maleate buffered sodium bicarbonate, buffer control containing nonbicarbonated Tris-maleate buffer, and a deionized water control was prepared. The final cell concentration in all tubes was approximately 7,5 × 10^5^ cells/mL using a 0.5 Mcfarland standard. The suspension in each tube was mixed by vortexing. Triplicate 20 *μ*L samples of each strain were then placed on a microscope slides and coverslips put on top to create microculture chambers. The slides were incubated in humid prewarmed Petri dishes at 37°C for 3 hours. The enumeration of the average germ tube positive cells for each bicarbonate concentration and controls was done after incubation. This allowed for the determination of the optimum bicarbonate concentration.

### 2.5. Serum Germ Tube Technique

Triplicate sets of test tubes containing 0.5–1.0 mL of pooled human serum were inoculated with 2-3 colonies of each isolate. The tubes were inoculated at 37°C for 3 hours after which a drop of each suspension was placed on labeled microscope slides for examination of germ tubes.

### 2.6. Evaluation of Rate of Germ Tube Formation

Bicarbonate slide microcultures in triplicate were prepared using the determined optimum 20 mM buffered bicarbonate concentration. Twelve microcultures were prepared also in triplicate for each of the 100* C. albicans* isolates and control strains. Twelve pooled serum tubes in triplicate were also inoculated with each isolate in parallel for comparison. Incubation was done at 37°C and after each 10-minute interval, the 3 microculture slides for each isolate and its respective 3 serum tubes were withdrawn and quickly enumerated for germ tube production. The time taken for each isolate to produce germ tubes was recorded.

### 2.7. Effect of CO_2_ on Rate of Serum Induced Germ Tube Formation

A panel of 12 test tube cultures was prepared for each isolate and control strains. The panel sets were incubated aerobically at 37°C. Simultaneously another set of 12 culture tubes for each isolate and control were incubated in 5–10% CO_2_ in candle jars. A set of tubes was also prepared and sealed air-tight. The tubes from CO_2_-free air, 5–10% CO_2_ enriched air, and air-tight tubes were withdrawn every 10 minutes without replacement and examined for germ tube formation.

### 2.8. Data Analysis

Results obtained from observations made on the capability and effect of bicarbonate concentrations, pooled human serum, and CO_2_ on germ tube formation by* C. albicans* were subjected to the Student *t*-distribution for paired samples. This statistical method was ideal for testing the hypotheses for significant differences in test results whereby *x*^*d*^ is mean difference in germ tube induction time; *s*^*d*^ is standard deviation of time differences; SEM *x*^*d*^ is standard error of the mean difference; *n* is sample size; *n* − 1 is degrees of freedom (d.f.). The confidence interval was set at 95% and therefore a significance level of 5% (0.05). In our sets of experimental assays, the relevant results were evaluated as follows:

- *t*-test for paired samples to test for any significant difference in the rate of germ tube formation between* C. albicans* isolates incubated in NaHCO_3_ against those incubated in pooled human serum
- *t*-test for paired samples to test for any significant difference in the rate of germ tube formation between* C. albicans* isolates incubated in human serum in normal air and air enriched with 5–10% CO_2_
- *t*-test for paired samples to test for any significant difference in the rate of germ tube formation in* C. albicans* isolates between each of three separate trials, with double trials each time, in testing the consistency of the human serum and bicarbonate techniques, respectively

## 3. Results

A total of 146 out of 150 (97.6%) isolates were germ tube positive when grown in foetal bovine serum. These were confirmed as* C. albicans.* All presumptive* C. albicans* species produced visibly much shorter germ tubes after incubation in buffered bicarbonate medium than those observed in pooled human serum cultures after 3 hours of incubation. Even after overnight incubation, there was an insignificant increase in germ tube development in buffered bicarbonate only cultures but extensive elongation of germ tubes into mycelial networks in pooled human serum cultures. Germ tube formation in buffered bicarbonate increased with increasing concentrations of sodium bicarbonate (Table 1). The results in Table 1 show that germ tube formation increased sharply from 0.0 mM NaHCO_3_ to 10.0 mM NaHCO_3_. The optimum concentration was found to be 20 mM. No germ tube formation occurred in buffer alone or in water controls. In Tris-maleate buffer alone, only scanty (21%) of cells showed minute budding and triplicate buffer control samples required scanning at least 5 fields to encounter any minute evidence of budding. No germ tubes were observed in Tris-maleate buffer control alone without bicarbonate or in deionized water alone. The NaHCO_3_ solutions shown in Table 1 were made in Tris-maleate buffer.

The time taken for observable initiation of germ tube formation varied from 20 to 100 minutes in both pooled human serum and sodium bicarbonate. The results for the mean time for germ tube formation are shown in Table 2. The samples in 20 mM bicarbonate buffer were not incubated in parallel with a similar set in CO_2_ enriched air because bicarbonate is considered to be the equivalent of CO_2_. Conversely, CO_2_ is a source for soluble bicarbonate where the atmosphere is enriched with it.

**Figure 1:**
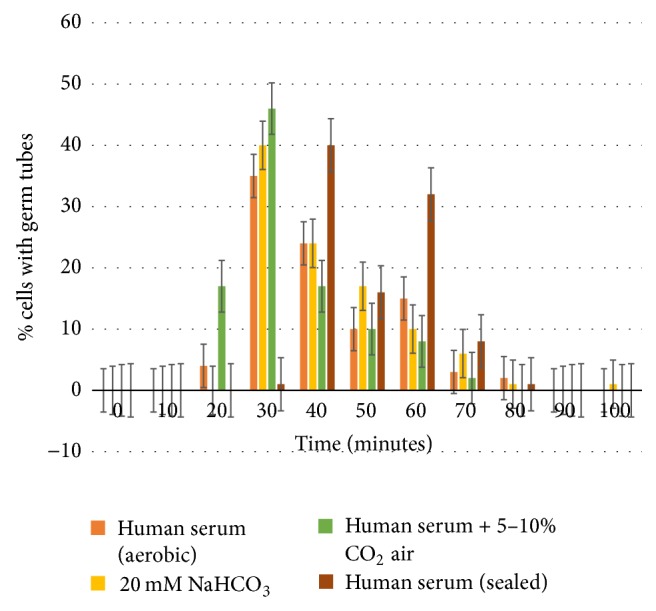
Plotted graph showing the proportions of cells forming germ tubes when incubating in 20 mM NaHCO_3_ (in air), human serum sealed and air-free, human serum in CO_2_-free air, and human serum in 5–10% CO_2_ enriched air.

Statistical values were used in the tests for paired samples to compare the rates of germ tube formation in bicarbonate and pooled human serum. The analysis for our results from experimental test sets was as follows: (a) *t*-test for paired samples to test for any significant difference in the rate of germ tube formation between isolates incubated in NaHCO_3_ against those in pooled human serum (*x*^*d*^ = 1.8, *s*^*d*^ = 11.84, *n* = 100, d.f. = 99, and SEM *x*^*d*^ = 1.184): The critical *t*-value of two tailed test is 1.96. Our calculated *t*-value was 1.52. This result showed evidence that there was no significant difference in the rates of germ tube induction mean time between pooled human serum and bicarbonate. (b) *t*-test for paired samples to test for any significant difference in the rate of germ tube formation between* Candida* isolates incubated in pooled human serum in normal air as opposed to 5–10% CO_2_ enriched air (*x*^*d*^ = 5.6, *s*^*d*^ = 13.05, *n* = 100, d.f. = 99, and SEM *x*^*d*^ = 1.305): This was a one-sided test so a critical *t*-value equals 1.645; hence our *t*-value was 5.6/1.305 = 4.29. Since this was above the critical 1.645 value, our results suggested that there was an increase in the rate of germ tube induction when isolates were incubated in 5–10% CO_2_. (c) *t*-test for paired samples to test for any significant difference in the rate of germ tube induction in* Candida* between each of 3 separate trials, with duplicate trials each time, to show whether there was consistency in the test results of induction using pooled human serum and bicarbonate techniques: (1) T1 versus T2 (*x*^*d*^ = 0.5, *s*^*d*^ = 4.97, *n* = 20, d.f. = 19, and SEM *x*^*d*^ = 1.111): hence *t* = 1/1.111 = 0.450. (2) T1 versus T3 (*x*^*d*^ = 1, *s*^*d*^ = 8.31, *n* = 20, d.f. = 19, and SEM *x*^*d*^ = 1.858): hence *t* = 1/1.858 = 0.538. (3) T2 versus T3 (*x*^*d*^ = 1.5, *s*^*d*^ = 7.92, *n* = 20, d.f. = 19, and SEM *x*^*d*^ = 1.771): hence *t* = 1.5/1.771 = 0.847. These were two tailed tests where *n* = 20, the d.f. = *n* − 1 = 19 with a critical *t*-value = 2.093 for analysis of the 3 double runs above. Since *t*-values obtained were all less than the critical value, there is no significant statistical evidence at 5% level to suggest a difference in the rate of germ tube induction in all pooled human serum trial runs. Similarly, the consistency of bicarbonate in inducing germ tube formation was tested in 3 dual runs: (1) T1 versus T2 (*x*^*d*^ = 1.5, *s*^*d*^ = 7.26, *n* = 20, d.f. = 19, SEM *x*^*d*^ = 2.133): hence *t* = 1.5/1.623 = 0.924. (2) T1 versus T3 (*x*^*d*^ = 3, *s*^*d*^ = 9.54, *n* = 20, d.f. = 19, and SEM *x*^*d*^ = 2.133): hence *t* = 3/2.133 = 1.406. (3) T2 versus T3 (*x*^*d*^ = 1.5, *s*^*d*^ = 11.08, *n* = 20, d.f. = 19, and SEM *x*^*d*^ = 2.478): hence *t* = 1.5/2.478 = 0.605. Since all 3 dual test runs showed *t*-values less than the critical 2.093, there was no significant statistical evidence at 5% level to suggest a difference in the rate of germ tube induction in these separate bicarbonate trial runs (Figure 1).

Statistical analysis of the results obtained from comparing the consistency of human pooled serum induction and 20 mM sodium bicarbonate induction of* C. albicans* morphogenesis and *t*-test of paired samples showed that there was a significant evidence (*p* < 0.05) to suggest consistency in results from both techniques.

## 4. Discussion

This study results showed that sodium bicarbonate alone can induce germ tube formation in a significant proportion of the* C. albicans* yeast cells (67%) in an inoculum. The capacity to induce germ tube formation by bicarbonate was found to be influenced by the concentration of bicarbonate [6–8]. The greatest increase in induction was observed between 0 mM and 10 mM bicarbonate (0–61% increase). A much smaller rise of 6% was observed at 20 mM bicarbonate, which was taken to be the optimum concentration for use in further tests to elaborate on induction. The 5% decline in germ tube test induction observed at 30 mM suggested a negative impact on germ tube formation with increases in bicarbonate concentration. We observed that evidence of inhibition of germ tube induction at a slightly increasing rate shown by a gradual decline in percentages of cells with visible germ tubes at higher bicarbonate concentration up to 50 *μ*M. The capability of bicarbonate alone to induce germ tube formation was suggested to be a function of the intracellular concentration of CO_2_ [12]. We examined this phenomenon by preparing parallel runs of human serum inoculated with yeast isolates and incubated aerobically, in tubes sealed air-tight and some tubes in carbon dioxide enriched air. The results showed clearly that samples incubated in carbon dioxide enriched air had a sizeable percentage (17%) of cells showing germ tubes after 20 minutes of incubation followed by isolates in serum incubated in normal air without CO_2_. Sims [12] suggested that CO_2_ could somehow drive the tricarboxylic acid cycle which drives energy for morphogenesis, which is an essential process in induction of* Candida* germ tubes [4, 12]. This was supported by our observation that deionized water alone without carbon dioxide failed to induce germ tube formation whereas bicarbonate without organic nutrients did. Tris-maleate buffer alone showed only 21% of yeast cells developing germ tubes. This observation needs to be investigated further in more detail, in light of the fact that maleate is an intermediate in the tricarboxylic acid cycle and could have possibly initiated germ tubing here.

Mock and coworkers [10] suggested that CO_2_ supply prior to germ tube formation resulted in abortive germination. Our results contradict this observation in that 5–10% carbon dioxide enriched air showed the earliest induction of germ tubes within 20 minutes but there were no germ tubes in serum exposed to air or air-tight serum tubes. It appeared that the percentage of germ tubing cells drastically decreased after 30 minutes of incubation under all our test conditions. We could not attribute this to an effect of carbon dioxide in light of this.

Our study showed no significant difference in mean time for observed induction of germ tubes between human serum and bicarbonate [8]. Although both media showed yeast germ tubes forming within 30 minutes, those in human serum were 3–5 times longer after 3 hours of incubation. Bicarbonate in normal human serum is found at a level of 20–26 mM which is consistent with our finding that the optimum concentration of bicarbonate germ tube induction was 20 mM. Our results from comparing bicarbonate and human serum (in air and air-tight conditions) suggest that bicarbonate might be the critical component in human serum for germ tube induction.

## Figures and Tables

**Table 1:** The capability and effect of bicarbonate on germ tube formation in *C. albicans.*

[NaHCO_3_] (mM)	B/C	0	10	20	30	40	50
Average% germ tube positive cells	21	0	61	67	62	53	45
Standard deviation	6.5		7.5	7.4	3.9	5.0	7.3

*Key*. B/C = buffer control only (no bicarbonate). 0 mM = deionized water only.

**Table 2:** Time taken (minutes) for observable induction of germ tubes in 100 *C. albicans* isolates when incubated in NaHCO_3_ against human serum aerobically, under 5–10% CO_2_ and sealed human serum.

Time (minutes)	Human serum (aerobic)	20 mM NaHCO_3_	Human serum (5–10% CO_2_ air)	Human serum (sealed)
0	0	0	0	0
10	0	0	0	0
20	4	0	17	0
30	35	40	46	1
40	24	24	17	40
50	10	17	10	16
60	15	10	8	32
70	3	6	2	8
80	2	1	0	1
90	0	0	0	0
100	0	1	0	0
